# Hydrocolloids from the Mushroom *Auricularia heimuer*: Composition and Properties

**DOI:** 10.3390/jof9060681

**Published:** 2023-06-16

**Authors:** Liudmila Kalitukha, Roman Bleha, Andriy Synytsya, Janina Kraska, Miriam Sari

**Affiliations:** 1 Good Feeling Products GmbH, 41468 Neuss, Germany; 2 Department of Carbohydrates and Cereals, Faculty of Food and Biochemical Technology, University of Chemistry and Technology, 16628 Prague, Czech Republic; roman.bleha@vscht.cz (R.B.); sinicaa@vscht.cz (A.S.); 3 Competence Center for Applied Mycology and Environmental Studies, Niederrhein University of Applied Sciences, 41065 Moenchengladbach, Germany; janina.kr@hotmail.de (J.K.); miriam.sari@hs-niederrhein.de (M.S.)

**Keywords:** *Auricularia heimuer*, extract, hydrocolloids, polysaccharides, composition, stability, moisture retention, sustainable production

## Abstract

The ear- to shell-shaped fruiting bodies of the genus *Auricularia* are widely used as food and in traditional medicinal remedies. This study was primarily focused on the composition, properties and potential use of the gel-forming extract from *Auricularia heimuer*. The dried extract contained 50% soluble homo- and heteropolysaccharides, which were mainly composed of mannose and glucose, acetyl residues, glucuronic acid and a small amount of xylose, galactose, glucosamine, fucose, arabinose and rhamnose. The minerals observed in the extract included approximately 70% potassium followed by calcium. Among the fatty and amino acids, 60% unsaturated fatty acids and 35% essential amino acids could be calculated. At both acidic (pH 4) and alkaline (pH 10) conditions, the thickness of the 5 mg/mL extract did not change in a temperature range from −24 °C to room temperature, but decreased statistically significantly after storage at elevated temperature. At neutral pH, the studied extract demonstrated good thermal and storage stability, as well as a moisture retention capacity comparable to the high molecular weight sodium hyaluronate, a well-known moisturizer. Hydrocolloids that can be sustainably produced from *Auricularia* fruiting bodies offer great application potential in the food and cosmetic industries.

## 1. Introduction

The genus *Auricularia* producing the peculiar ear- to shell-shaped jelly and slightly crunchy fruiting bodies is well known as a source of tasty food and pharmacologically active compounds. A large number of publications, as summarized in two recent reviews, ref. [1,2] show the anticancerogenic, antimicrobial, anti-inflammatory, antioxidant, anticoagulant, hypoglycemic and many other effects of *Auricularia* spp. The fungus has a history of more than 2000 years in China [3]. In 2018–2019, it was estimated as the second most cultivated mushroom worldwide, with 21% of the global mushroom production [4].

Like other jelly fungi, *Auricularia* fruiting bodies contain a high level of polysaccharides. They can be divided into homo- and heteropolymers. The homopolysaccharides have mostly a β-(1→3)-linked D-glucopyranosyl backbone with (1→6)-linked β-D-glucopyranosyl single groups or chains [5]. This type of β-glucans is the most abundant in edible and medicinal mushrooms as, inter alia, water-insoluble cell wall structural compounds. Interestingly, *Auricularia* neutral polysaccharides exist as single stiff comb-branched chains in water, whereas other soluble glucans (scleroglucan, schizophyllan, and lentinan) adopt a triple-helical conformation [5].

The heteropolysaccharides (often called (1→3)-α-D-glucuronoxylomannans or acidic polysaccharides) have (1→3)-linked α-D-mannopyranosyl (sometimes (1→4)-linked β-D-glucopyranosyl) backbones with the branches of D-glucopyranosyluronic acid, D-mannopyranose, D-glucopyranose, D-xylopyranose, D-galactopyranose residues, and *O*-acetyl groups [1,6,7]. Recently, chitosan with a high degree of deacetylation was also extracted from *Auricularia* [8]. Particular features of the *Auricularia*-soluble polysaccharides are their thermal stability and gel-forming properties in water. Because of the excellent swelling and water storage capacities [6], some extracts from *A. auricula-judae* and *A. polytricha* are offered as humectants, skin conditioning and skin protecting cosmetic ingredients [9]. 

Generally, hydrocolloids, which dissolve in water as colloids and show a high capacity for gel formation, have been used as gelling agents, thickeners and stabilizers in the food and cosmetics industry for decades. Industry demand for new hydrocolloids from natural sources is very high. Factors to be considered include but are not limited to manufacturing simplicity, scalability, costs, and economic use of the product.

The type and activity of the isolated compounds strongly depend on the species or strains and extraction process [10]. The spectrum of bioactivity of the *Auricularia* extracts is broad by the crude extracts and narrowed down after fractionation. As published in Elkhateeb et al. [11], the nonpolar n-hexane extract from *A. auricula-judae* was the most potent in cytotoxicity against the colon cancer HCT116 cell line, and the polar extracts showed antioxidant and antidiabetic activities. Another study of different fractions extracted from *A. auricula-judae* showed that, in contrast to the crude or neutral polysaccharides, the acidic polysaccharides had no impact on genetically diabetic KK-A^Y^ mice [12].

In the present work, the sustainably produced gel-forming crude extract from *Auricularia heimuer* fruiting bodies was tested for its composition, water retention capacity and storage stability at different concentrations, temperatures and pH values.

## 2. Materials and Methods

### 2.1. Extract Preparation and Deacetylation

Dried fruiting bodies of the mushroom were obtained on the market and identified by sequencing the nuclear ribosomal internal transcribed spacer region as *A. heimuer* [13]. This species is especially widespread in China and was incorrectly identified as *A. auricula* and *A. auricula-judae* for a very long time [14].

Gel-forming crude *Auricularia* extract was obtained by a patented method that included milling of the mushrooms and alcohol/water extraction followed by centrifugation and lyophilization using a VaCo freeze dryer (Zirbus Technology GmbH, Bad Grund, Germany) [15]. The yield of the dried extract did not exceed 10%. For analytical purposes, the crude extract was treated with aqueous ammonia at 37 °C to remove *O*-acetyl groups [16].

Different concentrations of the crude extract (1–5 mg/mL) were prepared by dissolving the lyophilized extract in sterile water under stirring (IKA Werke GmbH & Co. KG, Staufen im Breisgau, Germany) for 10 min followed by incubation in a water bath (P-D Industriegesellschaft mbH) at 100 °C for 5 min.

### 2.2. FTIR and NMR Measurements

FTIR spectra were measured on a Nicolet 6700 FTIR spectrometer in KBr pellets. The wavenumber range was 400–4000 cm^−1^ with a resolution of 2.0 cm^−1^ and 64 scans. The spectra were recorded, smoothed and baseline corrected using Omnic 8.0 software (Thermo Fisher Scientific, Waltham, MA, USA). Then, the FTIR spectra were exported in ASCII format to Origin 6.0 software (OriginLab Corporation, Northampton, MA, USA) for the creation of graphical output.

Proton NMR and ^13^C APT NMR spectra of the crude and deacetylated extracts were recorded on a Bruker Avance III HD 600 MHz (Bruker, Billerica, MA, USA) in D_2_O and D_2_O/NaOD solutions at 20 °C. Correlation ^1^H, ^1^H COSY, ^1^H, ^13^C HMQC and ^1^H, ^13^C HMBC NMR experiments were used for signal assignment. The 1D and 2D NMR spectra were processed using MestReNova 10.0 software (Mestrelab Research, Santiago de Compostela, Spain).

### 2.3. Determination of Total Carbohydrates and Glucans

The total carbohydrate content was determined spectrophotometrically using the anthrone–sulfuric acid assay [17] with some modifications. An anthrone solution was prepared with concentrated sulfuric acid (2 mg/mL). A 50 μL sample was mixed with 2 mL of the anthrone solution and incubated at 95 °C for 10 min. All samples were measured at 620 nm using an Eppendorf BioSpectrometer (Eppendorf, Wesseling, Germany) against a blank consisting of anthrone solution and distilled water. Glucose was used for calibration.

Total glucans, α- and β-glucans were determined using an enzyme-based assay developed for mushrooms and yeasts (Megazyme International Ireland Ltd., Wicklow, Irland), according to the manufacturer’s instructions [18]. To determine the total glucans, the samples (90 mg) were mixed with 2 mL of ice-cold 12 M sulfuric acid and incubated in an ice-water bath for 2 h. After 10 mL of distilled water was added, the samples were incubated in a boiling water bath for 2 h. After a neutralization step with 6 mL of 8 M sodium hydroxide, the samples were adjusted to 100 mL with sodium acetate buffer (200 mM, pH 4.5). Then, 0.1 mL aliquots were incubated with exo-1.3-β-glucanase (20 U/mL) and β-glucosidase (4 U/mL) at 40 °C for 60 min. Three milliliters of glucose oxidase/peroxidase (GOPOD) were added to each tube and incubated at 40 °C for 20 min.

To determine the α-glucan content, samples (100 mg) were stirred with 2 mL of 1.7 M sodium hydroxide on ice for 20 min. After adding 8 mL of sodium acetate buffer (1.2 M, pH 3.8) and 0.2 mL of invertase-amyloglucosidase mix (1630 U/mL and 500 U/mL), the samples were incubated in a water bath at 40 °C for 30 min. Then 0.1 mL aliquots were mixed with 0.1 mL of sodium acetate buffer (200 mM, pH 4.5) and 3 mL of GOPOD and incubated at 40 °C for 20 min. A yeast standard and an internal mushroom powder standard were used for the control. All samples were measured at 510 nm using an Eppendorf BioSpectrometer against a reagent blank. The β-glucan content was determined by subtracting the α-glucans from the total glucans.

### 2.4. Carbohydrate Profile

Acid hydrolysis with sulfuric acid, according to Sluiter et al. [19], followed by high-performance anion exchange chromatography coupled with pulsed amperometric detection (HPAE-PAD) was used to estimate glucose, mannose, glucuronic acid, xylose, galactose, rhamnose and arabinose. In short, a Dionex ICS-5000 ion chromatography system (Thermo Fisher Scientific, Waltham, MA) equipped with a CarboPac-PA20 guard column (3 × 30 mm) and a CarboPac-PA20 analytical column (3 × 150 mm) was used. An integrated pulsed amperometric detection (IPAD) with a gold working electrode and an Ag/AgCl reference electrode was applied. A standard carbohydrate quadruple potential waveform was used. The gold electrode was regularly maintained. Integration was performed using a Dionex Chromeleon 7.2 SR5 chromatography data system (ThermoFisher Scientific, Waltham, MA, USA). Elution was carried out with sodium hydroxide at a flow rate of 0.6 mL/min. The analyte concentration was calculated using a calibration curve.

Additionally, neutral polysaccharides were analyzed after hydrolysis in 72% H_2_SO_4_ as alditol acetates using gas chromatography coupled with flame ionization (GC-FID). In short, a Shimadzu GC-2010 (Shimadzu, Kyoto, Japan) equipped with a 30 m capillary column DB-225 with an internal diameter of 0.25 mm and a film thickness of 0.15 μm was used. The injector and detector temperatures were 220 °C and 230 °C, respectively. The oven temperature was kept at 200 °C for 1 min, then increased to 220 °C at a rate of 40 °C min^−1^ and maintained constant for 7 min. Afterwards, the temperature was elevated to 230 °C at a rate of 20 °C min^−1^ and maintained for 1 min, with a total run time of 9 min. 

The amount of glucosamine was estimated after hydrolysis with 6 N hydrochloric acid for 7 h at 100 °C according to Ekblad and Näsholm [20], followed by HPAE-PAD as described above.

Acid hydrolysis with sulfuric acid followed by high-performance liquid chromatography with refractive index detection (HPLC-RI) was used to estimate the amount of the acetyl groups. Briefly, the Series 200 high-performance liquid chromatography system with refractive index detector (PerkinElmer Life and Analytical Sciences, CT, USA), equipped with Phenomenex ^®^ Rezex™ ROA-Organic Acid H + (8%), column (300 × 7.8 mm) and TotalChrom 6.3.1 software (PerkinElmer Life and Analytical Sciences, CT, USA) was used. Elution was carried out with 5 mN sulfuric acid at a flow rate of 0.6 mL/min and column temperature of 65 °C. The analyte concentrations were calculated using an internal standard calibration method.

### 2.5. Lipid Fraction

Crude fat was determined as the sum of ethanol-extractable material, such as waxes, resins, and lipids, according to Sluiter et al. (procedure NREL/TP-510-42619) [21]. Samples were extracted with 95% ethanol using a Soxhlet extractor for 16 h. The extract was evaporated to dryness in a weighed flask using a vacuum evaporator at 80 °C and measured gravimetrically.

The fatty acid profile was measured using gas chromatography, according to German Society of Fats Science procedure DGF C-VI 10a (00) + 11f (08) after extraction with petroleum ether [22]. In brief, an Agilent DB-WAX column (60 m × 0.32 mm; film thickness: 0.5 μm; Agilent Technologies, Santa Clara, CA, USA) was used. The inner coating of this column consists of highly polar polyethylene glycol. Hydrogen was the carrier gas (0.95 mL min^−1^). The oven temperature was increased from 100 to 190 °C at a rate of 5 °C min^−1^ and kept for 14 min. Afterwards, the temperature was elevated to 250 °C at a rate of 5 °C min^−1^ and maintained constant for another 14 min. The flame ionization detector was operated at 260 °C. The samples (1 μL) were injected in the split mode (50:1). The analytical standards were obtained from Merck.

### 2.6. Protein Content

The proteinogenic amino acids (AAs) were measured after hydrolysis using high-pressure liquid chromatography (HPLC) with a fluorescence detector, according to the method of Algermissen et al. [23]. Briefly, hydrolysis of the samples was performed in 6 M hydrochloric acid at 110 °C for 18 h. For tryptophan analysis, hydrolysis was performed with lithium hydroxide (110 °C, 24 h). For methionine and cysteine analysis, the samples were pretreated with a mixture of formic acid and hydrogen peroxide (4 °C, 16 h) followed by hydrolysis with 6 M hydrochloric acid. After hydrolysis of the samples, the acid was removed using vacuum rotation evaporation. The residues were re-dissolved with water. Before HPLC, the AAs aspartic acid, glutamic acid, serine, histidine, glycine, threonine, arginine, alanine, tyrosine, valine, phenylalanine, isoleucine, leucine, lysine, methionine and cysteine were derivatized with phthalaldehyde; proline and hydroxyproline were derivatized with 4-chloro-7-nitrobenzofurazan. Tryptophan was directly measured using its own fluorescence. HPLC was performed using an Agilent system with a fluorescence detector, an RP-C18-column and a gradient method (methanol/acetate buffer). Elution was carried out at a flow rate of 0.5 mL/min at 37 °C for 45 or 10 min (for the samples derivatized with phthalaldehyde or 4-chloro-7-nitrobenzofurazan, respectively). L-Homoserine (4.44 ng) was used as an internal standard.

Proteins were recorded as a sum of the proteinogenic AAs after HPLC measurement. Additionally, the crude proteins were calculated using total nitrogen measured using the method of Kjeldahl [24]. The conversion factor of 4.16 was used, as recommended for fungi containing nonprotein nitrogen [25].

### 2.7. Ash Content and Minerals

The ash content was detected gravimetrically after incineration at 550 °C according to Sluiter et al. (NREL/TP-510-42622) [26], as well as using a Phoenix Black muffle furnace (CEM GmbH, Kamp-Lintfort, Germany). The sodium, potassium, calcium, magnesium, copper, iron and zinc contents were measured with atomic absorption spectrometry, according to the German Institute for Standardization standard (DIN EN 1134:1994-12) after pressure digestion (DIN EN 13805:2014) [27,28].

### 2.8. Relative Density and Flow Rate Measurements

The relative density, also called specific gravity (SG), is the measured density of a sample divided by the density of water at a certain temperature. In our experiments, the sample density at 20 °C was divided by the water density at the same temperature (SG_20/20_) using a digital density meter based on the oscillating U-tube principle (Anton Paar GmbH, Graz, Austria).

The consistency of the samples was compared using the custom-made analog of the Bostwick consistometer. A Bostwick consistometer is a simple instrument to determine the flow rate of different liquids using the distance of the fluid traveled along an inclined ruled track in the unit of time [29,30]. Consistency or thickness, which is a response of the liquid to gravity, should not be confused with viscosity, which is a measure of the resistance to flow. Nevertheless, depending on the type of samples, a strong to middle correlation of these parameters can be observed [31].

Two hundred microliters of each sample were applied to the starting point. A digital timer was started, and the sample was then free to flow through the 250 mm trough inclined at 45 °C. A reading of the distance (in mm) that each sample flowed was taken after 5 s. Measurements were made at room temperature (24 °C) and carried out in triplicate.

### 2.9. Moisture Retention Capacity

The moisture retention capacity was measured using a modified version of the method proposed by Li et al. [32]. Briefly, 10 mg of 2 mg/mL solution were added to a 9-cm^2^ filter paper 401 (VWR, Langenfeld, Germany) at 22 °C and 40% relative humidity. The weight change of the filter paper was recorded every minute to estimate the kinetic of water loss over time.

### 2.10. Statistical Analysis

Data represent the mean ± SD. Statistical analysis was performed with the GraphPad Prism 9.0 software package (La Jolla, CA, USA). Significant differences between the samples were determined using two-way analysis of variance (ANOVA). Significant values were defined as *p* < 0.05.

## 3. Results and Discussion

### 3.1. Composition

**Figure 1:**
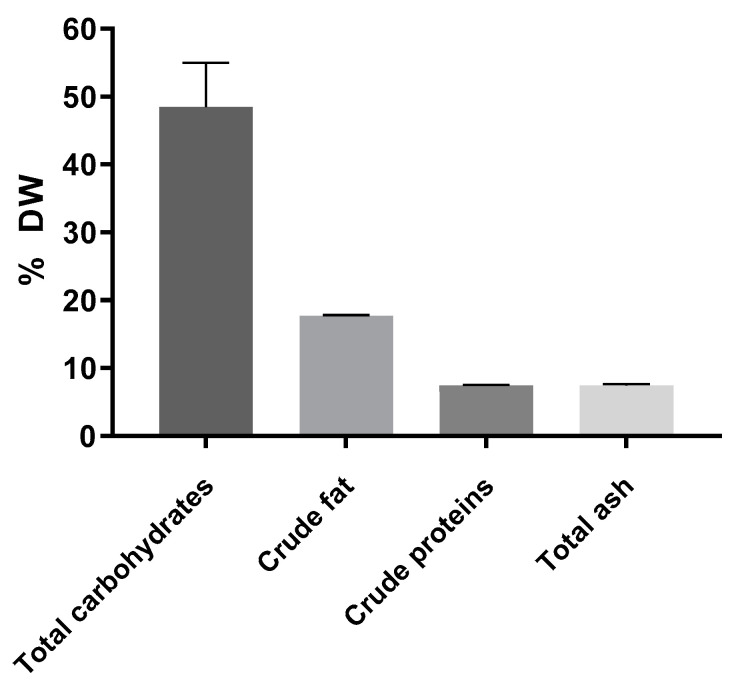
Composition of the *A. heimuer* extract (*n* = 3).

The proximate composition of the freeze-dried *A. heimuer* extract included approximately 50% soluble carbohydrates measured using the anthrone method, 17.7% crude fat, 7.4% proteins calculated using total nitrogen measured using the method of Kjeldahl with the conversion factor of 4.16 as recommended for fungi [25], and 7.4% ash (Figure 1). According to the published data, the composition of *Auricularia* strongly depends on the species, but carbohydrates are always the major compound [1,2].

**Figure 2:**
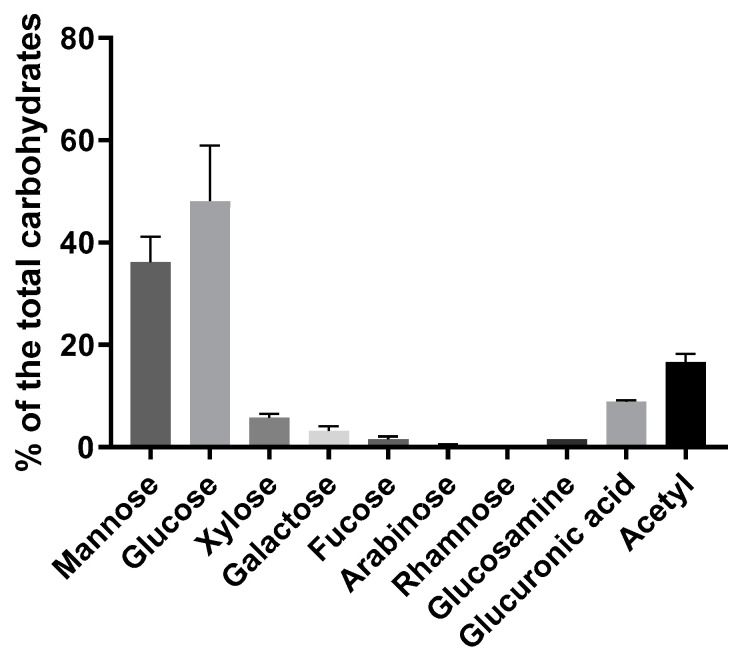
Carbohydrate composition of the *A. heimuer* extract (*n* = 3) calculated as a percentage of the total carbohydrates measured by anthrone method.

The carbohydrate composition of the *A. heimuer* extract, calculated as a percentage to the total carbohydrates, showed a majority of the mannose and glucose (Figure 2). Essential amounts of the acetyl groups and glucuronic acid (16.6 and 8.9%, respectively) were also detected. Approximately 10% was composed of the sum of the xylose, galactose, fucose, arabinose and rhamnose. All measured monomers are characteristics for the acidic heteropolysaccharides with mannopyranosyl backbones and branches of D-glucopyranosyluronic acid, D-mannopyranose, D-glucopyranose, D-xylopyranose, D-galactopyranose residues and *O*-acetyl groups, as well as for some other neutral sugars reported in *Auricularia* mushrooms earlier [33,34,35,36]. The acidic heteropolysaccharides are claimed to be responsible for the gel-forming properties of the *Auricularia* [1,6,37], as well as other jelly fungi, such as different *Tremella* species [5] or encapsulated yeast *Cryptococcus* [38]. Some amount of the soluble chitin/chitosan is also expected because of the availability of the glucosamine and acetyl groups. A high amount of the glucose monomers indicates the presence of homopolysaccharides (Figure 2). Indeed, approximately 20% of β-glucan was measured using an enzyme-based assay (Table 1). De facto, the β-glucan amount could be a bit lower due to part of the glucose belonging to heteropolymers. β-Glucans are typical to fungi and possess, inter alia, immunomodulatory, antibacterial and antitumor properties [39]. Usually presenting in a water-insoluble form, β-glucans are soluble in the studied *Auricularia* extract. However, the treatment with NH_4_OH led to the product being insoluble in hot water but soluble in aqueous alkaline solutions (see NMR analysis).

Proteins are the second most valuable but generally understudied compounds of *Auricularia* extract with their tolerance to acids, alkali, heat, freezing and dehydration [40]. In total, 8.2% of 18 proteinogenic amino acids were measured with HPLC in our experiments (Table 2).

Along the spectrum, more than 35% of essential amino acids and more than 60% of essential and conditionally essential amino acids could be calculated. The top five, or one half, of the amino acids comprised tyrosine, aspartic, glutamic, alanine and threonine. Some of the proteins and peptides isolated from *Auricularia* appear to have medical properties similar to polysaccharides, such as immunomodulation [40,41], or broad antimicrobial activity towards Gram-positive bacteria (*Staphylococcus aureus* and *Bacillus subtilis*), Gram-negative bacteria (*Escherichia coli, Pseudomonas aeruginosa,* and *Klebsiella pneumoniae*), yeast (*Candida albicans*) and dermatophytic pathogens (*Trichophyton schoenleinii, Trichophyton mentagrophytes, Microsporum gypseum*, and *Microsporum ferugineum*) [42]. The distinction of the effective component (protein or carbohydrates) has not yet been completely clarified. For example, the Tris and hot water extracts of the *A. auricular-judae* mushroom, studied by Oli et al. [42], contained 23.75% proteins and approximately 40% carbohydrates. Because of the already known antimicrobial activity of the carbohydrates [43,44], the effects of the extracts could refer to both compounds.

The crude fat proportion was 17.7% of the dried extract (Figure 1), or approximately 1.8% if calculated to the mushroom dry weight, and includes ethanol-extractable lipids, such as fatty acids, sterols, phospholipids, mono-, di- and triglycerides. Around one-tenth of the crude fat (1.6% of the extract DW) was composed of fatty acids, including more than 60% of unsaturated ones (Table 3). Approximately 90% of top four were represented by linoleic (C 18:2), oleic (C 18:1), palmitic (C 16:0) and stearic (C 18:0) fatty acids. These data are in accordance with the published average fat content of the *Auricularia* spp. that ranges from 0.5 to 4.5% with prevalence of linoleic and other unsaturated fatty acids [1,2].

The obtained ash content of 7.4% (Figure 1) was in the range between 1.1 and 9.4% earlier reported for different *Auricularia* spp. [2]. Approximately one half of the ash amount was composed of oxygen due to the formation of oxides during complete combustion. The rest of the residue on ignition contained minerals. The most abundant element was potassium (approximately 70%) followed by calcium, copper, magnesium, sodium and traces of iron and zinc (Table 4). A similar order of minerals with a prevalence of potassium was previously obtained [2]. A surprisingly high amount of copper in our extract could be explained by the nature of the substrate used during mushroom growing. Usually the amount of Cu is lower than Zn but could be increased by different supplements. For example, Yao et al. [45] reported that corn stalks as a cultivation substitution material increased protein, ash, copper and iron contents in *A. auricula* but reduced the content of magnesium, manganese, zinc and colloidal substances.

### 3.2. Spectroscopic Analyses

**Figure 3:**
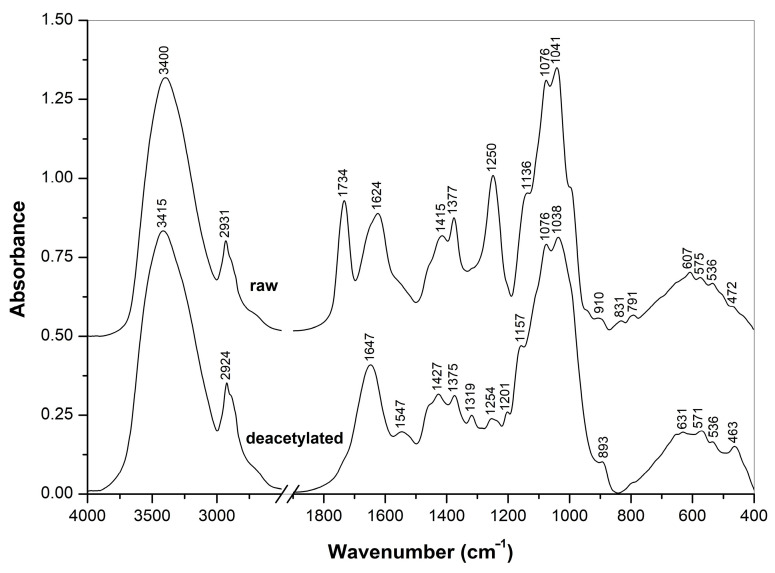
FTIR spectra of raw and deacetylated *A. heimuer* extracts.

**Figure 4:**
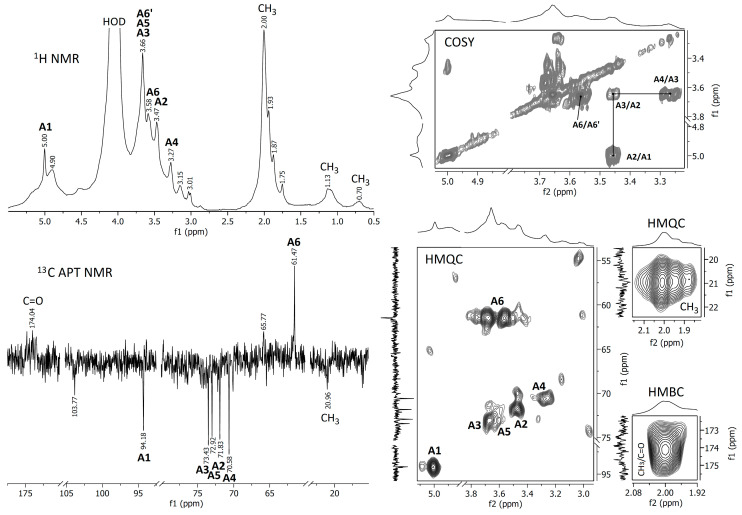
NMR spectra of the raw *A. heimuer* extract.

**Figure 5:**
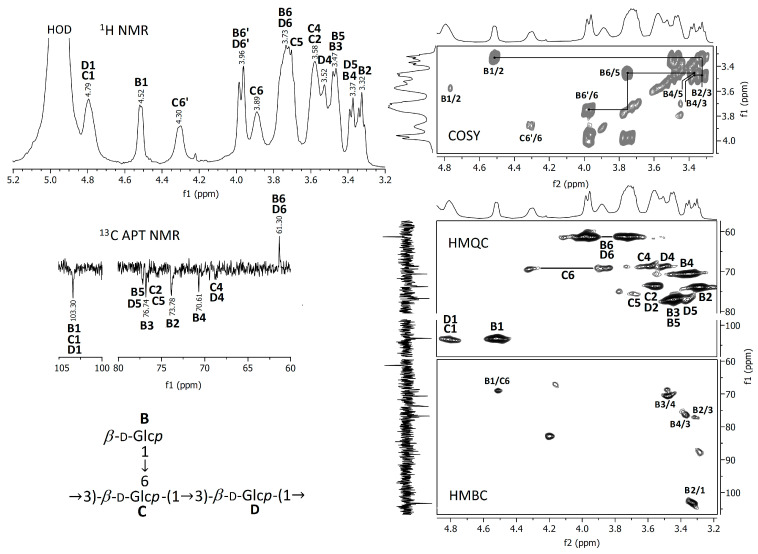
NMR spectra of the *A. heimuer* extract after the treatment with NH_4_OH.

In order to better evaluate the chemical composition, the crude and deacetylated extracts were analyzed using FTIR and NMR spectroscopic methods. The spectra obtained are represented in Figure 3, Figure 4 and Figure 5.

#### 3.2.1. FTIR Spectra

FTIR spectra of the crude and NH_4_OH-treated *Auricularia* extracts measured in the KBr tablets (Figure 3) showed significant differences in their composition. The strong IR bands at 1734 and 1250 cm^−1^ observed for the crude extract were assigned to the C=O and C-O-C stretching vibrations of *O*-acetyl groups, respectively, and two bands at 1624 and 1415 cm^−1^ arose from the stretching vibrations of carboxylate anions from the salts of carbonic acids, possibly uronic acids as part of polysaccharides [46,47,48]. These *O*-acetyl and carboxylic groups are probably originated from glucuronoxylomannan, which was previously isolated from this mushroom [1,6,7,35], from mushroom *Tremella aurantialba* [49] and yeast *Cryptococcus neoformans* [38,50,51,52]. All these bands disappeared after the treatment with ammonium. By contrast, the characteristic bands of β-D-glucan at 1377, 1076 and 1041 cm^−1^ were pronounced in the spectrum of the crude *Auricularia* extract, and these and other β-D-glucan bands at 1375, 1157, 1038 and 893 cm^−1^ were presented in the extract after the NH_4_OH treatment [53,54]. Therefore, according to the FTIR spectra, the crude extract contained β-D-glucans and possibly also *O*-acetylated glucuronoxylomannan. The treatment with NH_4_OH led to *O*-deacetylation and removal of the heteropolysaccharides by the washing with aqueous ethanol, while β-D-glucan was retained after this treatment.

#### 3.2.2. NMR Spectra

Proton NMR and ^13^C APT NMR spectra of the crude and NH_4_OH-treated *Auricularia* extracts are demonstrated in Figure 4 and Figure 5; the zooms of some 2D NMR spectra (COSY, HMQC and HMBC) are also shown in these figures. A strong proton signal at approximately 2 ppm, together with smaller ones at 1.93, 1.87 and 1.75 ppm and corresponding carbon signals around 21 ppm indicate the *O*-acetylation of monosaccharide units in the crude extract [35]. This assignment is confirmed by the HMQC signal at 2.0 ppm/21.0 ppm and the HMBC signal at 2.0 ppm/174 ppm, indicating interactions between CH_3_ protons and CH_3_ and C=O carbons in *O*-acetyls, respectively. The proton signals at 5.00, 3.66, 3.58, 3.45 and 3.27 ppm and the carbon signals at 94.18, 73.43, 72.92, 71.83, 70.58 and 61.47 ppm were assigned to 1,3-linked α-D-mannopyranose units (A), which probably compose the backbone of glucuronoxylomannan. Unfortunately, the low resolution of the HMBC spectrum does not permit to assign possible *O*-acetylation patterns for this carbohydrate unit. The main proton and carbon signals observed for the *Auricularia* extract after the treatment with NH_4_OH were assigned to terminal, 1,3,6-linked and 1,3-linked β-D-glucopyranosyl units designed as units B, C and D (Figure 5). These signal confirmed the presence of highly branched (1→3)(1→6)-β-D-glucan having terminal β-D-glucopyranosyls attached to the O-6 position of some 1,3-linked β-D-glucopyranoses in the backbone. Branched β-D-glucan of the same structure has been described earlier for *Auricularia* mushrooms [55].

### 3.3. Storage Stability

The storage stability of the samples was tested by measuring their flowability at different concentrations (1–5 mg/mL), temperatures (−26 °C, 4 °C, RT and 40 °C), and pH values of 4, 6.7 and 10.

In some tests, the high molecular weight sodium hyaluronate was used as a reference, as its MW, which is more than 1 MDa, is comparable with polysaccharides of *Auricularia,* [1] and it has a reputation as an optimal moisture retention ingredient [56,57].

#### 3.3.1. Consistency at Neutral pH

**Figure 6:**
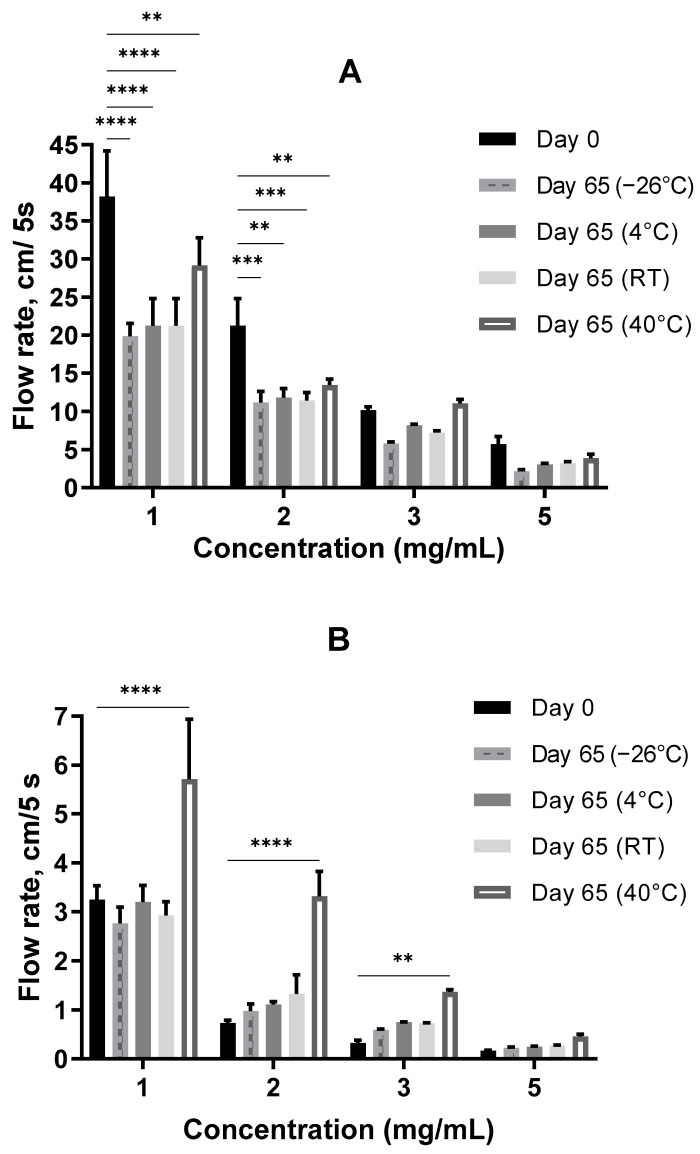
The flowability of the *A. heimuer* (**A**) and sodium hyaluronate (**B**) solutions (pH = 6.3 ± 0.3) after a 2-month storage at different temperatures. The asterisks represent the significance at Day 0 vs. Day 65: ** *p* < 0.01, *** *p* < 0.001, **** *p* < 0.0001, two-way ANOVA followed by Dunnett’s post hoc test.

**Figure 7:**
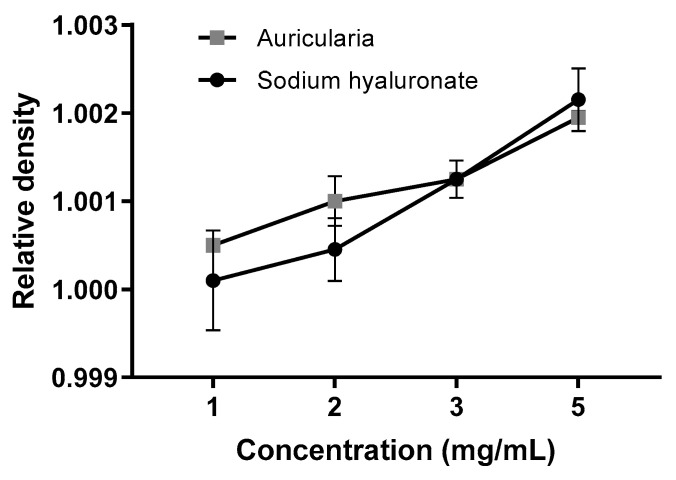
Relative densities of the *A. heimuer* and sodium hyaluronate samples at different concentrations at the beginning of the storage period (*n* = 3).

The flow rate of the samples, measured with a custom-made analog of the Bostwick consistometer, was changed after 2 months of storage at a neutral pH = 6.3 ± 0.3. At the start point (Day 0), the sodium hyaluronate hydrogel was approximately 10–30 times thicker as *Auricularia* at the same concentrations (Figure 6A,B, Day 0). This is not surprising, because the dissolved hyaluronic acid could form a three-dimensional cellular structure at concentrations less than 1 µg/mL [58], whereas other biopolymers can make pseudo-gels only when concentrations are equal to or above 10 mg/mL [57]. To ensure that the initial differences in the consistency of the *A. heimuer* and sodium hyaluronate samples were not caused by the different densities, the relative densities of the samples were measured. As expected, this parameter positively correlated with increases in the concentration from 1 to 5 mg/mL solution, but at the same concentration the relative densities of the *Auricularia* and sodium hyaluronate samples were not distinguishable (Figure 7).

As shown in Figure 6A, the flow rate of the *A. heimuer* samples decreased with the length of time of storage at different temperatures. At lower concentrations of 1 and 2 mg/mL, the differences were statistically significant, whereas at the higher concentrations of 3 and 5 mg/mL a decreasing tendency was observed.

On the contrary, an increasing tendency of the flowability, which means a decreasing thickness with the length of the storage time, was observed for the 2–5 mg/mL sodium hyaluronate solutions at all studied temperatures. At an elevated temperature of 40 °C, the increase in the flowability was especially remarkable (Figure 6B).

In this manner, the difference in the thicknesses of the *Auricularia* and sodium hyaluronate samples dropped by three times on average with the storage time.

#### 3.3.2. Consistency at Different pH Values

**Figure 8:**
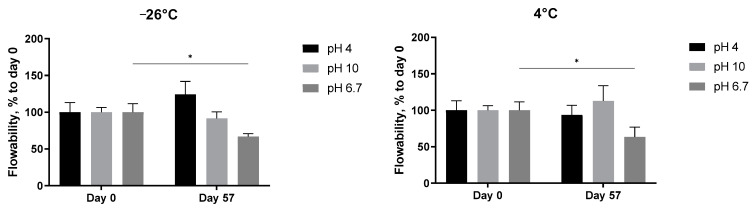

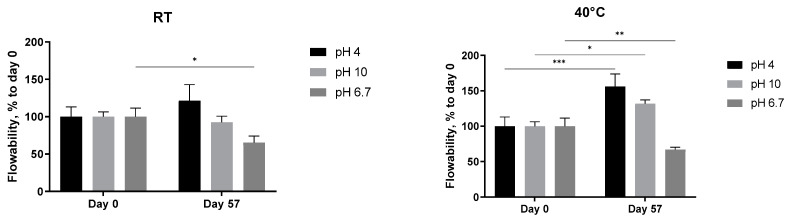
Effect of pH values on the flowability of the *A. heimuer* 5 mg/mL solution after storage at different temperatures (−24 °C, 4 °C, room temperature, and 40 °C). The asterisks represent the significance at Day 0 vs. Day 57: * *p* < 0.05, ** *p* < 0.01, *** *p* < 0.001. Two-way ANOVA followed by Šídák’s multiple comparisons test.

The pH-dependent stability of the 5 mg/mL *A. heimuer* solution after storage at different temperatures was also studied. Decreasing the flow rate of the extract at neutral pH, presented already in Figure 6, was observed again, but this time statistically significant (Figure 8, pH 6.7). At both acidic (pH 4) and alkaline (pH 10) conditions, the abovementioned increase in the thickness with the length in time of storage was not registered anymore. The flowability of the samples was not affected in the range from −24 °C to room temperature, but significantly increased at 40 °C (Figure 8). A similar negative impact of the extreme pH values on the viscosity of the *Auricularia* polysaccharide aqueous solutions was previously published and explained by the breakdown of the hydrogen bonds of the carboxyl group of glucuronic acid [7,37]. Bao et al. could show that, namely, hydrogen bonds rather than electrostatic interactions are the deciding force maintaining the *Auricularia* gel network [37]. An elevated temperature is suggested as an additional factor promoting the breakup of hydrogen bonds [59].

### 3.4. Moisture Retention Capacity

**Figure 9:**
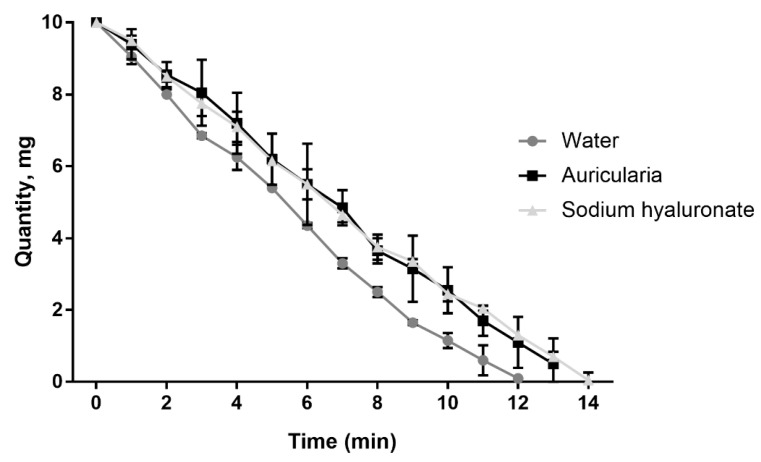
Kinetics of the moisture retention capacity of water, sodium hyaluronate and *A. heimuer* extract (*n* = 3).

The moisture retention capacity of the *A. heimuer* extract was as potent as that of high molecular weight sodium hyaluronate—an optimal and broadly used humectant (Figure 9). A water retention capacity similar to the sodium hyaluronate was measured earlier using an aqueous extract of the white strain of *A. fuscosuccinea* [60].

## 4. Conclusions

The particular advantage of the *Auricularia* gel-forming extract described here encompasses its possible environmentally friendly and resource-saving production with a good option to scale-up. The extract was composed of a large share of soluble homo- and heteropolysaccharides and included minerals, essential amino acids and unsaturated fatty acids. The thickness of the *A. heimuer* hydrogel was inferior to the high molecular weight sodium hyaluronate, but the difference decreased with the length of time of storage due to the higher stability of the *Auricularia* extract, especially at elevated temperature. The gel strength of the *A. heimuer* extract was diminished in acidic or alkaline solutions (pH 4 and 10). At neutral pH, the extract possessed good thermal and storage stability, as well as a moisture retention capacity comparable to the high molecular weight sodium hyaluronate, the well-known moisturizer. The hydrocolloids, sustainably produced from the *Auricularia* fruiting bodies, offer great application potential in many areas of the food and cosmetic industries. Their nutritional benefits and practical applications should be further explored.

## 5. Patent

Kalitukha L., Sari M., inventors; Lexut A., Lexut P., assignees. Gelbildende Extrakte aus den Pilzen der Gattung Ohrlappenpilze (*Auricularia*) sowie Verfahren zu deren Herstellung (Gel-Forming Extracts from the Fungi of the Genus *Auricularia* and Method for Their Preparation). Germany patent DE 102021104013A1. 19 February 2021. German.

## Figures and Tables

**Table 1:** Relative content of total glucans, α-glucans, and β-glucans in *A. heimuer* extract (*n* = 7).

Constituent	Mean ± SD (g/100 g DW)
Total glucans	18.77 ± 1.47
α-Glucans	0.53 ± 0.13
β-Glucans	18.86 ± 0.71

**Table 2:** Amino acid profile of *A. heimuer* extract (*n* = 3).

Amino Acids	Mean, g/kg	SD	% of Total Amount
Alanine	**6.6**	0.7	8
Arginine **	3.2	0.3	4
Aspartic acid	**9.0**	0.9	11
Cysteine **	1.4	0.1	2
Glutamic acid	**8.8**	0.9	11
Glycine **	3.9	0.4	5
Histidine *	2.4	0.2	3
Isoleucine *	2.6	0.3	3
Leucine *	5.4	0.5	7
Lysine *	2.5	0.2	3
Methionine *	0.9	0.1	1
Phenylalanine *	4.2	0.4	5
Proline **	3.6	0.4	4
Serine	5.5	0.5	7
Threonine *	**6.2**	0.6	8
Tryptophan *	1.1	0.1	1
Tyrosine **	**10.0**	1.0	12
Valine *	4.3	0.4	5
Total amount (18 AAs)	81.6		100
Essential AAs *	29.6		36
Essential * + semi-essential ** AAs	51.7		63
**Top 5 AAs**	40.6		50

* essential, ** semi-essential.

**Table 3:** Fatty acid profile of *A. heimuer* extract (*n* = 3).

Fatty Acid	Mean ± SD (% of Total Fatty Acid)
Capric acid (C 10:0)	0.06 ± 0.01
Lauric acid (C 12:0)	0.1 ± 0.01
Myristic acid (C 14:0)	0.57 ± 0.06
Pentadecanoic acid (C 15:0)	1.47 ± 0.15
Palmitic acid (C 16:0)	**21.76** ± 2.15
Palmitoleic acid (C 16:1)	0.48 ± 0.05
Margaric acid (C 17:0)	0.41 ± 0.04
Stearic acid (C 18:0)	**10.49** ± 1.04
Oleic acid (C 18:1)	**23.55** ± 2.33
Linoleic acid (C 18:2)	**34.05** ± 3.37
gamma-linolenic acid (C 18:3)	0.21 ± 0.02
alpha-linolenic acid (C 18:3)	1.09 ± 0.11
Arachidic acid (C 20:0)	1.18 ± 0.12
Eicosenoic acid (C 20:1)	0.23 ± 0.02
Eicosadienic acid (C 20:2)	0.15 ± 0.01
Eicosatrienic acid (C 20:3)	0.32 ± 0.03
Erucic acid (C 22:1)	2.97 ± 0.29
Docosadienic acid (C 22:2)	0.08 ± 0.01
Lignoceric acid (C 24:0)	0.84 ± 0.08
Unsaturated fatty acids	63
**Top 4**	90

**Table 4:** Mineral content of the *A. heimuer* extract (*n* = 3).

Mineral Content	Mean ± SD (mg/100 g DW)
Macroelements	
K	2560 ± 235
Ca	527 ± 52
Mg	203 ± 20
Na	92 ± 9
Microelements	
Cu	238 ± 24
Fe	11.5 ± 1.1
Zn	3.7 ± 0.4
Total	3635

## Data Availability

Not applicable.

## Abbreviations

AAs: amino acids; ANOVA, analysis of variance; APT, attached proton test; DW, dry weight; FTIR, Fourier-transform infrared spectroscopy; GC-FID, gas chromatography coupled with flame ionization; GOPOD, glucose oxidase/peroxidase; HPAE-PAD, high-performance anion exchange chromatography coupled with pulsed amperometric detection; HPLC, high-pressure liquid chromatography; IR, infrared; KBr, potassium bromide; NMR, nuclear magnetic resonance; SG, specific gravity.
